# Long-term safety and efficacy of Omnitrope®, a somatropin biosimilar, in children requiring growth hormone treatment: Italian interim analysis of the PATRO Children study

**DOI:** 10.1186/s13052-016-0302-3

**Published:** 2016-11-03

**Authors:** Lorenzo Iughetti, Gianluca Tornese, Maria Elisabeth Street, Flavia Napoli, Claudia Giavoli, Franco Antoniazzi, Stefano Stagi, Caterina Luongo, Sara Azzolini, Letizia Ragusa, Gianni Bona, Clara Zecchino, Tommaso Aversa, Luca Persani, Laura Guazzarotti, Emiliano Zecchi, Alberto Pietropoli, Stefano Zucchini

**Affiliations:** 1 Pediatric Unit, Department of Medical and Surgical Sciences for Mother, Children and Adults, University of Modena and Reggio Emilia, Via del Pozzo, 41124 Modena, Italy; 2 Institute for Maternal and Child Health IRCCS Burlo Garofolo, Trieste, Italy; 3 Department of Paediatrics- Arcispedale S. Maria Nuova-IRCCS, Reggio Emilia, Italy; 4 Pediatric Unit, Istituto Giannina Gaslini, Genova, Italy; 5 Endocrinology and Metabolic Diseases Unit, Department of Clinical Sciences and Community Health, Fondazione IRCCS Ca’ Granda Ospedale Maggiore Policlinico, Università degli Studi di Milano, Milano, Italy; 6 Pediatric Unit, Policlinico Giambattista Rossi, University of Verona, Verona, Italy; 7 Health Science Department, Anna Meyer Children’s University Hospital, University of Florence, Firenze, Italy; 8 Department of Woman, Child and General and Specialized Surgery, Seconda Università degli Studi di Napoli, Napoli, Italy; 9 Pediatric Endocrinology Unit, Department of Woman’s and Child’s Health, University Hospital of Padua, Padova, Italy; 10 UOC Pediatria, I.R.C.S.S. Oasi Maria SS, Troina, Italy; 11 Division of Paediatrics, Azienda Ospedaliero-Universitaria Maggiore della Carità, Novara, Italy; 12 Department of Sciences and Pediatric Surgery, University of Bari “A. Moro”, Bari, Italy; 13 Department of Human Pathology of Adulthood and Childhood, University of Messina, Messina, Italy; 14 Department of Clinical Sciences and Community Health, University of Milan, Milano, Italy; 15 Division of Endocrine and Metabolic Diseases, IRCCS Istituto Auxologico Italiano, Milano, Italy; 16 Department of Paediatrics, Paediatric Endocrinology Unit, University of Milan, Milan, Italy; 17 Sandoz S.p.A, Milan, Italy; 18 Hexal AG, Holzkirchen, Germany; 19 Unit of Pediatric Endocrinology, S.Orsola-Malpighi Hospital, Bologna, Italy

**Keywords:** Adolescents, Children, Infants, Omnitrope®, Paediatric, Recombinant human growth hormone

## Abstract

**Background:**

PATRO Children is an ongoing observational, longitudinal, non-interventional, global post-marketing surveillance study, which is investigating the long-term safety and effectiveness of Omnitrope®, a somatropin biosimilar to Genotropin®, in children with growth disturbances. The primary endpoint of PATRO Children is long-term safety and the secondary endpoint is effectiveness, which is assessed by analysing auxological data such as height (HSDS) and height velocity (HVSDS) standard deviation scores. Here, we report the data from the Italian interim analysis of PATRO Children data up to August 2015.

**Methods:**

PATRO Children is enrolling children who are diagnosed with conditions of short stature requiring GH treatment and are receiving Omnitrope®. Adverse events (AEs) are assessed in all Omnitrope®-treated patients. Height is evaluated yearly to near-adult (final) height, and is herein reported as HSDS; height velocity is also assessed and reported as a standard deviation score (HVSDS).

**Results:**

Up to August 2015, a total of 186 patients (mean age 10.2 years, 57.5 % males) were enrolled :156 [84 %] had growth hormone deficiency, 12 [6.5 %] were born small for gestational age, seven [3.8 %] had Prader-Willi syndrome, one [0.5 %] had Turner syndrome and one [0.5 %] had chronic renal insufficiency; seven [3.8 %] patients had other indication profiles. The mean treatment duration with Omnitrope® was 28.1 ± 19.1 months. AEs were reported in 35.6 % of patients and included headache, pyrexia, arthralgia, abdominal pain, leg and/or arm pain and increased blood creatine phosphokinase. Two serious AEs in two patients were thought to be drug-related; one patient experienced a minimal increase in a known residual craniopharyngioma, and another a gait disturbance with worsening of walking difficulties. Similar to investigational studies, Omnitrope® treatment was associated with improvements in both HSDS and HVSDS.

**Conclusions:**

Omnitrope® appears to be well tolerated and effective for the treatment of a wide range of paediatric indications, which is consistent with the outcomes from controlled clinical trials. These results need to be interpreted with caution until the data from the global PATRO Children study are available.

## Background

Over the last 30 years, several recombinant human growth hormone (rhGH) products have been approved for the treatment of children with growth disorders associated with short stature [1–7], and multiple studies, both observational and randomized, have established the efficacy and safety of rhGH products (also known as somatropin) [8–12].

Omnitrope® (Sandoz, Kundl, Austria), expressed by a transformed strain of *Escherichia coli*, was developed as the biosimilar medicinal product to the originator Genotropin® (Pfizer Limited, Sandwich, UK) and in 2006 was the first product to be approved by the European Medicines Agency (EMA) via the European biosimilar regulatory pathway. Omnitrope® is licensed for use in treating children with growth hormone deficiency (GHD) and also those with conditions that can affect growth such as Turner syndrome (TS), chronic renal insufficiency (CRI), Prader-Willi syndrome (PWS) and children born small for gestational age (SGA) [13].

Results from pivotal phase III studies of Omnitrope® have demonstrated that Omnitrope® is safe and effective in infants, children and adolescents with GHD [9, 14]; however, more real-world data of Omnitrope® in pediatric patients are required to consolidate the phase III outcomes in clinical practice. To address this, the PAtients TReated with Omnitrope® (PATRO) Children study was initiated. PATRO Children is a long-term, post-marketing surveillance (PMS) study investigating the safety and efficacy of Omnitrope® in children with growth disturbances. It has been conducted as part of the risk management plan for Omnitrope®, to fulfil the commitment with the EMA. Interim 1-year results of the patients included in this study up to September 2012 (*n* = 1837) have previously been reported [15] and annual updates have been presented at international meetings [16]. Up to January 2016, 4675 patients have been recruited from 291 sites across 14 countries (Austria, Czech Republic, France, Germany, Italy, Poland, Romania, Slovenia, Spain, Sweden, Taiwan, UK, Canada and USA) in the PATRO Children study. Herein we present the results of 186 patients recruited at 17 Italian sites, representing the subgroup of patients enrolled in Italy since the beginning of the study up to August 2015.

## Methods

### Study design

The design of this multicentre, open, longitudinal, non-interventional PMS study has been published previously in detail [15]. Briefly, this study was conducted in children’s hospitals and specialised endocrinology clinics in several of the countries where Omnitrope® was approved. Patients enrolled in the study are infants, children and adolescents receiving treatment with Omnitrope® for any diagnosis and who had written informed consent provided by their parents or legal guardian. Hormone-naïve patients and patients who had received a previous rhGH were both eligible for inclusion. The study was reviewed and approved by each study site’s Independent Ethics Committee/Institutional Review Board, and was conducted in accordance with the Oviedo Human Right Convention and the Declaration of Helsinki.

### Treatment and outcomes

Patients included in the PATRO Children study received Omnitrope® treatment in accordance with the recommendations in the Summary of Product Characteristics [13] and/or the prescribing information of the respective countries. The primary objective of this ongoing observational study is to collect and analyse the data on long-term safety of Omnitrope® in infants, children and adolescents treated within routine clinical practice, with particular emphasis on the following aspects: diabetogenic potential of rhGH therapy in children born SGA and treated for growth disturbance, occurrence of malignancies in rhGH treated patients, occurrence and clinical implications of anti-rhGH antibodies and the risks of rhGH treatment in patients with PWS.

All adverse events (AEs), serious adverse events (SAEs), adverse drug reactions (ADRs), and serious ADRs were recorded in electronic case report forms and entered into the Sandoz safety database. Laboratory values (including glucose metabolism) were also recorded at least once a year during the study.

The secondary objective was to collect and analyse data on the efficacy of the treatment. Efficacy endpoints include the auxological data height standard deviation scores (HSDS) and height velocity standard deviation scores (HVSDS), derived from height measurements [17, 18].

### Statistical analysis

In this interim analysis, statistical calculations were performed using the software package SAS version 9.3. AEs were coded using Medical Dictionary for Regulatory Activities (MedDRA) version 17.1. Concomitant medication was coded according to World Health Organization Drug Dictionary (version 14.3) and the medications were tabulated by Anatomical Therapeutic Chemical term in their current version. For continuous/quantitative variables, descriptive statistics, including the number of data values available, number of data values missing, arithmetic mean, standard deviation, minimum, median and maximum were calculated. When appropriate, continuous parameters were compared using *t*-tests or Wilcoxon non-parametric tests. For categorical/qualitative variables, frequency and percentage tables were generated. When appropriate, categorical data were compared using chi-square or Fisher’s exact tests. Statistical tests were two-sided at the significance level of 0.05.

## Results

Up to August 2015, 186 patients with a mean age of 10.2 years, including 57.5 % male, were enrolled at 17 sites in Italy (Table 1) and had received Omnitrope® treatment for a mean of 28.3 ± 19.1 months. Most patients (*n* = 156; 84.0 %) had GHD, 12 were born SGA (5.6 %), seven (3.8 %) had PWS, one (0.5 %) had CRI and another one (0.5 %) had TS; seven (3.8 %) patients were enrolled with other indication profiles. Overall, 89.8 % of patients were naïve to hormone therapy and Omnitrope® was prescribed as their first therapy. The mean duration of growth hormone pre-treatment for all remaining patients (10.2 %) was similar for all indications (27.8 ± 19.2 months), with the exception of one patient with PWS whose pre-treatment period was 47.3 months.

**Table 1:** Baseline characteristics and demographics of Italian patients enrolled in the PATRO Children study up to August 2015

Characteristic	*N* = 186
Gender(%)	
Male	107 (57.5)
Female	79 (42.5)
Chronological age, years	10.2 ± 3.3
HSDS ± SD	−2.29 ± 0.86 (N_miss_ = 30)
Height velocity, cm/year ± SD	3.9 ± 2.1 (N_miss_ = 91)
BMI, kg/m^2^ ± SD	17.2 ± 3.6 (N_miss_ = 35)
Diagnosis at presentation, *n* (%)	
GHD	156 (84.0)
SGA	12 (6.5)
TS	3 (1.6)
PWS	7 (3.8)
CRI	1 (0.5)
Other	7 (3.8)
Previous treatment status, *n* (%)	
Hormone naïve	167 (89.8)
Pre-treated	19 (10.2)
Omnitrope® dosing at baseline, mg/kg/day	0.032 ± 0.008
Duration of Omnitrope® treatment, months	28.3 ± 19.1

All values are presented as mean ± standard deviation unless otherwise stated

*BMI* body mass index, *CRI* chronic renal insufficiency, *GHD* growth hormone deficiency, *HSDS* Height Standard Deviation Scores, *N*_*miss*_ number of patients with data missing, *PWS* Prader-Willi syndrome, *SD* standard deviation, *SGA* small for gestational age, *TS* Turner syndrome

Eighty-five patients had discontinued documentation in PATRO Children study at the time of this analysis. Reasons for discontinuation included: patient reached final height/bone age maturation (*n* = 26; 30.6 %); switch to other growth hormone products (*n* = 21; 24.7 %; these patients, all coming from one site, were switched to other rhGHs, between November 2011 and January 2012, after the decision to interrupt data generation); lost to follow-up (*n* = 7; 8.2 %); other reasons (*n* = 6; 7.1 %); AEs (*n* = 2; 2.4 %); a slowdown of height velocity below 1 cm/year (*n* = 1; 1.2 %); patient did not wish to continue the injections (*n* = 1; 1.2 %) and patient non-compliance (*n* = 1; 1.2 %). The reason for discontinuation was unknown in the remaining 20 patients (23.5 % of patients).

### Safety

Up to August 2015, there were 142 AEs registered in the database occurring in 66 (35.6 %) of the 186 patients included in the Italian safety analysis set. The most common AEs reported (incidence > 9.12 over 438.8 patient-years) were headache (13 patients; 7.0 %; GHD *n* = 12, other *n* = 1), pyrexia (7 patients; 3.8 %; GHD *n* = 5, SGA *n* = 1, CRI *n* = 1), arthralgia (5 patients; 2.7 %; GHD *n* = 5), abdominal pain (4 patients; 2.2 %; GHD *n* = 2, SGA *n* = 1, other *n* = 1), leg and/or arm pain (4 patients; 2.2 %; GHD *n* = 3, other *n* = 1) and increased blood creatine phosphokinase (4 patients; 2.2 %; GHD *n* = 4). Nineteen ADRs occurred in 18 (9.1 %) patients; one patient experience two events of increased creatine phosphokinase levels. The number of ADRs with available MedDRA preferred terms was 17 (incidence > 2.28); these are summarized in Table 2. Two additional ADRs without available MedDRA preferred terms were reported in patients with GHD; one male patient had subclinical hyperthyroidism and one female had high insulin-like growth factor-1 (IGF-1) levels (667.4 ng/mL).

**Table 2:** Adverse drug reactions in the safety analysis set (*n* = 186)

Adverse drug reaction^a^,^b^	Patients, *n* (%)	Incidence (patient-years^c^ = 438.8)
Increased blood creatine phosphokinase	4 (2.2)	11.4
Impaired glucose tolerance	2 (1.1)	4.56
Craniopharyngioma	1 (0.5)	2.28
Drug administration error	1 (0.5)	2.28
Exostosis	1 (0.5)	2.28
Gait disturbance	1 (0.5)	2.28
Headache	1 (0.5)	2.28
Hyperinsulinism	1 (0.5)	2.28
Increased insulin-like growth factor	1 (0.5)	2.28
Metabolic disorder	1 (0.5)	2.28
Sleep apnoea syndrome	1 (0.5)	2.28
Snoring	1 (0.5)	2.28

^a^Preferred term/MedDRA dictionary

^b^Mild or moderate

^c^Until cut-off date

A total of 10 SAEs in eight (4.3 %) patients were reported. Among these, two SAEs in two GHD patients were considered possibly related to Omnitrope® treatment. One patient with previous craniopharyngioma (a 19-year-old male) experienced a minimal increase in the size of residual craniopharyngioma 5 years after initiation of Omnitrope®. For safety reasons, Omnitrope® was discontinued in this patient and restarted 4 months later; as the increase was not confirmed by a subsequent magnetic resonance imaging (MRI) scan, this event was reported as completely resolved. The second patient (an 8-year-old male), who had skeletal dysplasia and syndactyly, experienced a gait disturbance with a worsening of his walking difficulties. In this patient, Omnitrope® treatment was permanently discontinued and the outcome of the event was not reported.

To date, there have been no confirmed cases of type 1 or type 2 diabetes mellitus with Omnitrope® treatment; however, two patients had mildly impaired glucose tolerance. Similar to the global preliminary results, no growth hormone-related malignancies or investigator-reported data concerning anti-rhGH antibody titres have been reported. In patients with PWS, four AEs were reported in two patients; the first patient had one mild AE (bronchitis) and two SAEs (atelectasis, interstitial lung disease), whilst the second patient had sleep apnoea syndrome, which was considered to be drug-related.

### Efficacy

**Fig. 1:**
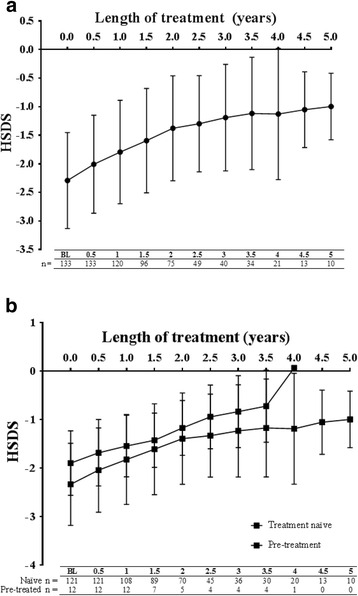
Change in height standard deviation scores (HSDS) in (**a**) the total efficacy analysis set and (**b**) pre-treated and naïve patients over 5 years. BL, baseline

**Fig. 2:**
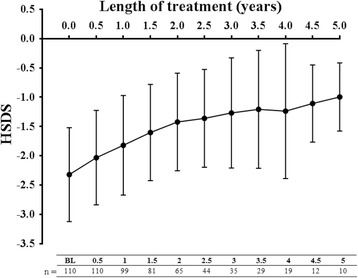
Change in height standard deviation scores (HSDS) in hormone-naïve patients with growth hormone deficiency (GHD) included in the efficacy analysis set over 5 years. BL, baseline

With Omnitrope® treatment, HSDS values improved gradually over time in the total patient population (Fig. 1a), from a mean HSDS value of –2.29 ± 0.84 (*n* = 133) at baseline to –1.13 ± 1.15 (*n* = 21) at Year 4. This positive trend continued to Year 5 (–0.10 ± 0.58 [*n* = 10]) and the improvement in HSDS occurred irrespective of pre-treatment status (Fig. 1b). A similar trend was observed in hormone-naïve patients with GHD (Fig. 2). Due to the small number of Italian patients with indications other than GHD, the data obtained was not statistically meaningful.

**Fig. 3:**
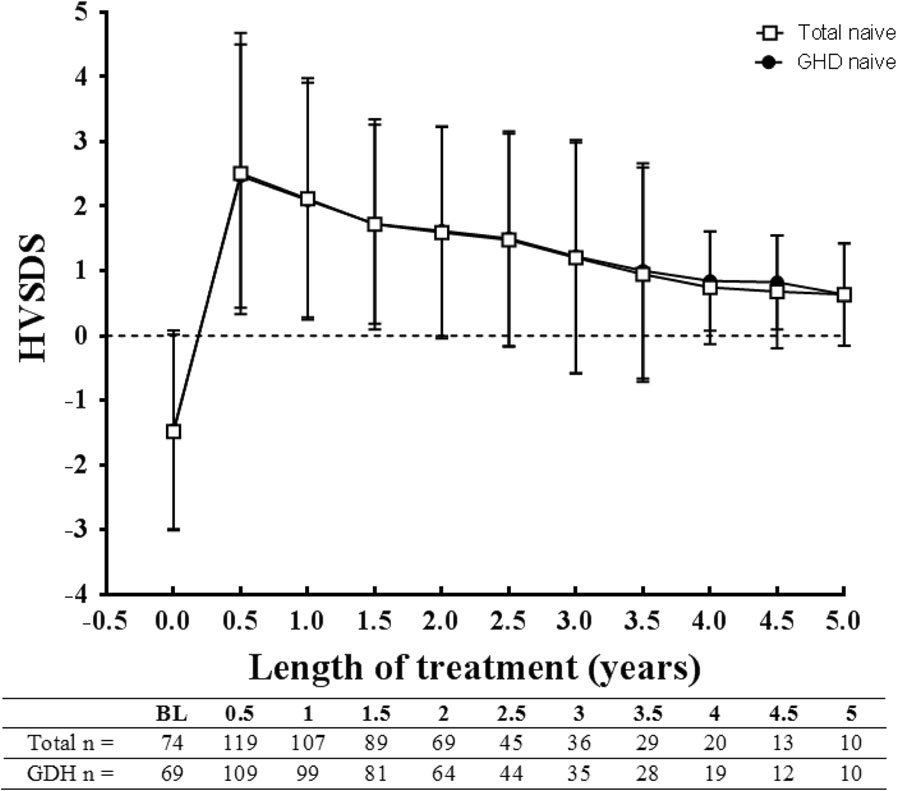
Change in height velocity standard deviation scores (HVSDS) in the total hormone-naïve patient population and in hormone-naïve patients with growth hormone deficiency (GHD) included in the efficacy analysis set over 5 years. BL, baseline

In hormone-naïve patients, mean HVSDS increased from a baseline value of –1.49 ± 1.50 (*n* = 74) to a peak mean of 2.50 ± 2.17 at 0.5 years (*n* = 119) and stabilised to 0.74 ± 0.87 at 4.0 years (*n* = 20) and 0.63 ± 0.79 at 5.0 years (*n* = 10; Fig. 3). This trend in HVSDS was also observed in hormone-naïve patients with GHD (Fig. 3) and children born SGA (from –1.79 ± 0.82 [*n* = 4] at baseline to 1.13 ± 1.23 at 1.5 years [*n* = 4]).

## Discussion

The results of this interim analysis showed that Omnitrope® was well tolerated and effective in Italian children with growth disturbances requiring rhGH treatment. The most common AEs reported were headache, pyrexia, arthralgia, abdominal pain and increased blood creatine phosphokinase. ADRs were reported in 9.1 % of patients and two of these were considered serious.

Some studies have suggested a link between rhGH treatment and the development of diabetes [19], whilst other studies have not established the same relationship [9, 20]. More specifically, children born SGA may be at increased risk of developing insulin resistance and type 2 diabetes [21] and there is concern that rhGH may amplify this risk. A study based on the Kabi Pharmacia International Growth Study (KIGS) database evaluating the adverse events of rhGH therapy in children with PWS reported that four patients developed hyperglycaemia and six patients had presumptive diabetes with therapy [22]. In accordance with our results, where no cases of type 1 or type 2 diabetes were reported, the findings from the Omnitrope® phase III clinical study, as well as the first data published from the global PATRO Children study, did not support a link between rhGH treatment therapy and diabetes [14, 15]; however, the potential association between rhGH treatment therapy and the development of diabetes still has to be confirmed in every day clinical practice.

There is conflicting evidence regarding whether there is an association between rhGH treatment therapy and the risk of developing cancer [23]. Concerns emerged from observations where growth hormone was shown to raise serum concentrations of IGF-1, as IGF-1exhibits mitogenic and anti-apoptotic properties *in vitro* as well as in animal models [24]. The occurrence of cancer as well as an increased risk of developing a second malignancy was observed in cancer patients treated with growth hormone compared with non-treated cancer patients [24]. However, a study based on the KIGS database reported no association between rhGH therapy and increased incidence of cancer [25]. In this interim analysis of Italian data from the PATRO Children study, one patient presented with an increase of a residual craniopharyngioma and treatment was temporarily interrupted, although a subsequent MRI scan did not confirm this increase. To further investigate the incidence of malignancies in this interim analysis, these findings will need to be compared with results from the global PATRO Children study. No other malignancies suspected to be related to the Omnitrope® treatment were reported, and at data cut-off, there have been no investigator-reported anti-rhGH antibody titres.

The efficacy data indicate a positive effect of Omnitrope® on growth parameters in paediatric Italian patients, confirming the outcomes of the phase III trials. Similar to investigational studies, Omnitrope® treatment was associated with an improvement in both HSDS and HVSDS in paediatric patients with GHD [9, 14]. As expected, patients who had received rhGH pre-treatment had a baseline HSDS higher than those who were naïve to rhGH, but the responses followed the same trend for the two populations.

A study based on the KIGS database reported a change in median HSDS in Caucasian patients with GHD from –2.4 to –0.8 in males and from –2.6 to –1.0 in females following rhGH replacement therapy [26]. These results are similar to the findings reported in this study, although a comparison is difficult due to the variation in treatment duration and baseline age. In fact, analysis of the KIGS database has confirmed an inverse correlation between age at treatment start and growth response [26].

The results of this analysis are consistent with those reported in the global PATRO Children study up to September 2012 [15]. These data from the PATRO Children study support that Omnitrope® is well tolerated in routine clinical practice; no confirmed cases of diabetes, no reports of malignancy and no additional safety issues were reported. However, considering the August 2015 cut-off date, there are some differences between the characteristics of the patients included in the full analysis of the PATRO Children study and the current analysis of the Italian patients. In the global analysis, 58.7 % of patients included are GHD, 25.9 % of the patients were born SGA and the remaining children are diagnosed with TS (4.4 %), PWS (2.3 %), CRI (0.7 %) or other diagnosis (6.0 %) [15]. In contrast, in this Italian analysis the proportion of patients with GHD is very high (~80 % of patients) whilst only 6.5 % of patients were born SGA. This may be an indication of a difference between how the children are diagnosed and/or treated in Italy compared with the other countries.

The results of this analysis are also consistent with the latest data of the overall PATRO Children study, which was analysed in January 2016 (data on file). In the 4675 patients recruited so far, the mean duration of Omnitrope® treatment has been 30.2 ± 22.0 months. Overall, 1653 patients (35.4 %) have experienced AEs and 248 (5.3 %) have experienced an SAE; SAEs were considered treatment-related in 22 (0.5 %) patients. One case of gradual onset of type 1 diabetes has been reported and there have been no reports of rhGH-related malignancies or additional safety concerns. Additionally, no clinically relevant positive anti-rhGH antibody titres have been reported. Efficacy data indicate that Omnitrope® has a positive effect on growth parameters in children: after 4 years of treatment, Omnitrope® resulted in significant improvements in growth parameters across all indications, irrespective of gender or pre-treatment status. In particular, over the first 4 years of treatment, greater height gains were observed in hormone-naïve patients, with a mean HVSDS (SD) of 1.66 (2.67) and ΔHVSDS +4.56 in patients with GHD (*n* = 411) and of 0.80 (2.21) and ΔHVSDS +3.41 in children born SGA (*n* = 231).

PWS is a rare condition and the PATRO Children study provides an opportunity to investigate how children with PWS respond to rhGH therapy [15]. In this interim analysis of Italian patients, one patient reported mild sleep apnoea syndrome, which was considered to be related to the rhGH therapy. Sleep apnoea has been reported as a cause of death in a PWS patient treated with growth hormone therapy in a study based on the KIGS database [22]. While rhGH therapy appears to be relatively well tolerated in the overall population of Italian children with PWS, these results need to be interpreted with caution due to the small patient number (*n* = 7) and will need to be confirmed by the global analysis of the PATRO Children study, when published.

While the results of this analysis are supportive of the long-term interventional trials of Omnitrope®, there are some limitations to the interpretation of these findings. The patients included in this analysis were mainly diagnosed as having GHD which, as mentioned earlier, is not representative of the results of the therapy in all different indications of rhGH treatment. However, we feel that this study is reflective of the real-world clinical practice in Italy and may be interpreted as such.

## Conclusions

This analysis showed that Omnitrope® is well tolerated and effective in a wide range of paediatric indications in routine clinical practice. The safety and efficacy of Omnitrope®, a somatropin biosimilar, was consistent with that observed in controlled clinical trials. All these data can contribute to ruling out the hypothetical concerns related to the use of biosimilars in real-world clinical practice.

## Abbreviations

ADR: Adverse drug reaction
AE: Adverse events
CRI: Chronic renal insufficiency
EMA: European Medicines Agency
GHD: Growth hormone deficiency
HSDS: Height standard deviation score
HVSDS: Height velocity standard deviation score
IGF-1: Insulin-like growth factor-1
KIGS: Kabi Pharmacia International Growth Study
MRI: Magnetic resonance imaging
PATRO: PAtients TReated with Omnitrope®
PMS: Post-marketing surveillance
PWS: Prader-Willi syndrome
rhGH: Recombinant human growth hormone
SADR: Serious adverse drug reaction
SAE: Serious adverse event
SGA: Small for gestational age
TS: Turner syndrome
